# Increased cardiac involvement in Fabry disease using blood-corrected native T1 mapping

**DOI:** 10.1038/s41598-023-31211-9

**Published:** 2023-03-17

**Authors:** Jannike Nickander, Ben Cole, Sabrina Nordin, Ravi Vijapurapu, Richard P. Steeds, James C. Moon, Peter Kellman, Martin Ugander, Rebecca Kozor

**Affiliations:** 1 Department of Clinical Physiology, Karolinska University Hospital, and Karolinska Institutet, Stockholm, Sweden; 2 Kolling Institute, Royal North Shore Hospital, and University of Sydney, Sydney, Australia; 3 Institute of Cardiovascular Science, University College London, London, UK; 4 Institute of Cardiovascular Science, University of Birmingham, Birmingham, UK; 5 National Heart, Lung, and Blood Institute, National Institutes of Health, Bethesda, MD USA; 6 Charles Perkins Center, Faculty of Medicine and Health, University of Sydney, Sydney, Australia; 7 Royal North Shore Hospital, North Shore Private Hospital, Sydney, Australia; 8 Sydney Medical School, University of Sydney, Sydney, Australia

**Keywords:** Cardiovascular biology, Cardiac hypertrophy, Cardiology, Medical research

## Abstract

Fabry disease (FD) is a rare lysosomal storage disorder resulting in myocardial sphingolipid accumulation which is detectable by cardiovascular magnetic resonance as low native T1. However, myocardial T1 contains signal from intramyocardial blood which affects variability and consequently measurement precision and accuracy. Correction of myocardial T1 by blood T1 increases precision. We therefore deployed a multicenter study of FD patients (*n* = 218) and healthy controls (*n* = 117) to investigate if blood-correction of myocardial native T1 increases the number of FD patients with low T1, and thus reclassifies FD patients as having cardiac involvement. Cardiac involvement was defined as a native T1 value 2 standard deviations below site-specific means in healthy controls for both corrected and uncorrected measures. Overall low T1 was 135/218 (62%) uncorrected vs. 145/218 (67%) corrected (*p* = 0.02). With blood-correction, 13/83 previously normal patients were reclassified to low T1. This reclassification appears clinically relevant as 6/13 (46%) of reclassified had focal late gadolinium enhancement or left ventricular hypertrophy as signs of cardiac involvement. Blood-correction of myocardial native T1 increases the proportion of FD subjects with low myocardial T1, with blood-corrected results tracking other markers of cardiac involvement. Blood-correction may potentially offer earlier detection and therapy initiation, but merits further prospective studies.

## Introduction

Fabry disease (FD) is a rare lysosomal storage disorder^1^ resulting in progressive sphingolipid accumulation in multiple organs including the heart^2^. Cardiac involvement is the leading cause of death in FD patients^3,4^. Initiation of early treatment seems to be favorable to avoid irreversible, progressive disease^5,6^, but identifying early cardiac involvement is challenging.

Myocardial sphingolipid accumulation can be detected by cardiovascular magnetic resonance (CMR) using native T1 mapping^7^. Low myocardial native T1 typically precedes the development of the classic sign of left ventricular hypertrophy (LVH)^8–10^, and thus can be an early marker of disease. Myocardial native T1 detects signals from both myocytes (with sphingolipid) and intramyocardial blood. Blood has a longer and more variable T1, and the contaminating signal acts as noise, reducing the accuracy and precision of myocardial T1 measurement for myocyte sphingolipid accumulation^11^. However, blood T1 can be independently measured in the cardiac blood pool, permitting a correction strategy^11,12^. It is currently unknown if blood-correction increases the proportion of patients with cardiac involvement in FD patients, and if blood-correction is clinically feasible across different sites and scanners.

We hypothesised that myocardial T1 blood-correction increases the proportion of FD patients with cardiac involvement, defined as low native T1, in FD patients. Therefore, the aim of this image-based study was to investigate if blood-correction of myocardial native T1 increases the number of FD patients with low myocardial T1 compared to controls.

## Methods

### Study population

An international multicenter study, gene-positive adult FD patients (*n* = 218) were recruited from the Fabry400 study (Clinical Trials.gov identifier NCT03199001, registered 26/06/2017), which consisted of patients in Sydney, Australia, London, UK, and Birmingham, UK. Healthy controls (*n* = 117) with no history of cardiovascular or kidney disease, were included from the same sites in Sydney (*n* = 20), Birmingham (*n* = 20), and London (*n* = 77). Consecutive patients (*n* = 200) referred for diagnostic CMR at Karolinska University Hospital, Stockholm, Sweden, were included for blood-correction model construction. Ethical approval was granted by site-specific local ethics review boards. All participants provided informed consent.

### Image acquisition

CMR was conducted at 1.5 T (Avanto (UK), Aera (Australia and Sweden), Siemens Healthcare, Erlangen, Germany), using anterior and posterior phased-array surface coils with the participants lying supine. A midventricular native myocardial T1 map was acquired using an electrocardiographically-gated modified Look-Locker inversion recovery (MOLLI) sequence with a 5 s(3 s)3 s sampling scheme and the T1 map reconstructed using in-line motion correction^13^. Typical imaging parameters included SSFP single-shot readout in end-diastole, flip angle (FA) 35 degrees, pixel size 1.4 × 1.9 mm^2^, slice thickness 8.0 mm, imaging duration 167 ms, time to echo (TE) 1.12 ms, matrix size = 256 × 144 and field of view (FOV) 360 × 270 mm^2^.

CMR scans assessing left ventricular (LV) function used retrospectively ECG gated balanced SSFP cine imaging covering the entire LV in short-axis slices, and three long-axis slices. Typical imaging parameters included FA 68º, pixel size 1.4 × 1.9 mm^2^, slice thickness 8.0 mm, TR /TE = 37.05/1.19 ms, matrix size = 256 × 144 and FOV 360 × 270 mm^2^.

LGE images were acquired using phase sensitive inversion recovery sequence with ECG gating following an intravenous contrast bolus (0.1 mmol/kg, Gadoterate meglumin, Dotarem, Guerbert S.A, France). Typical CMR parameters for LGE included: slice thickness 8 mm, pixel size 1.3 × 1.3 mm, time to repetition (TR) 8 ms, TE 3.2 ms, inversion time (TI) 300 ms and FA 25°.

### Image analysis

T1 maps from patients and healthy volunteers were analyzed with either Segment software version 2.0 R5152 (Medviso AB, Lund, Sweden) or CVI42 (Circle Cardiovascular Imaging, Calgary, Canada) as previously described^11^, by one experienced observer per site. In brief, myocardial native T1 values in the mid septum and blood T1 values from both the LV and RV cavities were obtained from manually drawn regions of interest (with care taken to avoid blood pool contamination, papillary muscles, and trabeculations, as well as focal abnormalities in the myocardium). A linear correlation between myocardial and blood measurement was assumed. Myocardial T1 (1/R1) was corrected for blood T1 according to the formula: R1_corrected_ = R1_uncorrected_ + constant (X_mean_ − X_patient_), where X was the mean blood R1 (1/T1 average LV + RV blood), and the constant was the slope from the linear regression  of myocardial R1 and mean R1 blood. The data for the formula was acquired from the Swedish patient cohort for model construction, as previously described^11^.

LV volumes, ejection fraction and myocardial mass were quantified using the dedicated software. Body surface area (BSA) was calculated according to Mosteller^14^. Volumetric measurements and myocardial mass were indexed to BSA. LV mass was quantified including papillary muscles and LVH defined as increased LV myocardial mass according to age and sex matched normal reference ranges^15^. LGE images were read for the presence or absence of LGE.

### Statistical analysis

Continuous variables were reported as means together with their standard deviation (SD). Ordinal variables were reported as percentages. Pearson’s linear correlation was used to define the slope between myocardial T1 and blood measurements. Low native T1 in FD patients was defined as a native myocardial T1 value 2 standard deviations below the mean in healthy volunteers at that site, both for corrected and uncorrected measures. The proportion of patients with sphingolipid accumulation before and following correction was evaluated for differences with McNemar’s test. Mean values were compared using paired or unpaired t-tests as appropriate after being assessed for normality using the Kolmogorv-Smirnov test. The relationships between uncorrected and blood-corrected native T1 was assess with linear correlation and data expressed as *r*. Statistical analysis was performed using Microsoft Excel (Microsoft, Redmond, Washington, USA) and IBM SPSS Statistics (IBM SPSS Statistics 25, IBM, New York, USA). Significance was defined as *p* < 0.05.

### Ethics, consent and permission

All study procedures were carried out in accordance with relevant guidelines and regulations as per the Declaration of Helsinki and Good Clinical Practice for involving human participants. The study was approved by the Swedish Ethical Review Authority, Dnr 2022-03123-01 and all patients provided written informed consent.

## Results

### Study population

Baseline characteristics for FD patients are shown in Table 1. There were a total of 218 gene-positive adult FD patients (age mean ± SD 46 ± 15 years, 54% females) included from Sydney (*n* = 42, age 47 ± 16 years, 52% females), London (*n* = 129, age 45 ± 14 years, 61% females) and Birmingham (*n* = 47, age 47 ± 15 years, 36% females). There were 117 healthy volunteers, Table 2, (age 46 ± 15 years, 50% females, p = 0.94 compared to FD patients) included from Sydney (*n* = 20, age 29 ± 5 years, 50% females), Birmingham (*n* = 20, age 51 ± 15 years, 50% females), and London (*n* = 77, age 49 ± 14 years, 51% females). Model construction used 200 consecutive patients (age 51 ± 18 years, 50% females) referred for clinical or research CMR in Stockholm.

**Table 1:** Baseline characteristics of FD patients.

Characteristic	Total (*n* = 218)	Females (*n* = 118)	Males (*n* = 100)
Female sex, *n* (%)	118 (54)		
Age, years	46 ± 15	45 ± 14	46 ± 16
Height, cm	169 ± 9	166 ± 8	173 ± 10
Weight, kg	71 ± 14	70 ± 14	72 ± 14
BSA, m^2^	1.8 ± 0.2	1.8 ± 0.2	1.9 ± 0.2
LVEDV, ml	129 ± 34	121 ± 23	138 ± 41
LVEDVI, ml/m^2^	71 ± 15	68 ± 11	74 ± 19
LVESV, ml	36 ± 18	32 ± 13	41 ± 21
LVESVI, ml/m^2^	20 ± 9	18 ± 7	22 ± 10
LVSV, ml	93 ± 23	89 ± 16	97 ± 29
LVSVI, ml/m^2^	51 ± 11	50 ± 8	52 ± 14
LVEF, %	73 ± 9	74 ± 8	71 ± 19
LVM, g	162 ± 78	135 ± 59*	193 ± 86
LVMI, g/m^2^	88 ± 39	75 ± 30	104 ± 42
Hematocrit	40 ± 4	39 ± 3†	42 ± 4‡
On ERT, %	47%*	42%	59%*

Data presented as mean ± SD.

*BSA* body surface area, *ERT* enzyme replacement therapy, *LVEDV* left ventricular end diastolic volume, *LVESV* left ventricular end systolic volume, *LVSV* left ventricular stroke volume, *LVEF* ejection fraction, *LVM* left ventricular mass, *I* signifies indexed to BSA.

*Data missing for *n* = 3, ^†^*n* = 30 and, ^‡^*n* = 39 patients.

**Table 2:** Native T1 values in FD patients and healthy volunteers.

Cohort	Uncorrected native T1 (ms)	Blood-corrected native T1 (ms)	LV blood T1 (ms)	RV blood T1 (ms)
All FD patients	932 ± 62	922 ± 59	1633 ± 91	1607 ± 93
Female FD patients	953 ± 63	939 ± 55	1652 ± 89	1624 ± 93
Male FD patients	907 ± 51	901 ± 58	1610 ± 88	1586 ± 89
Healthy volunteers	1013 ± 41	1009 ± 35	1580 ± 105	1600 ± 91
Female healthy volunteers	1026 ± 42	1015 ± 36	1630 ± 98	1616 ± 108
Male healthy volunteers	1000 ± 36	1003 ± 34	1569 ± 72	1543 ± 87
Consecutive patients	1030 ± 43	1030 ± 38	1594 ± 90	1540 ± 106

Data presented as mean ± SD.

*FD* Fabry disease, *LV* left ventricle, *RV* right ventricle.

### Native T1 and blood-correction

All native T1 data is presented in Table 2. The resultant correction formula was: Myocardial R1_corrected_ = R1_uncorrected_ + 0.493 (0.000639694 − R1 blood_patient_). Native myocardial T1 was 932 ± 62 ms for the total cohort of FD patients, and following blood-correction 922 ± 59 ms, *p* < 0.001. Site-specific cutoff values for a low and high native T1 are shown in Table 3.

**Table 3:** Site-specific cutoff values for uncorrected and corrected native T1.

Site	Uncorrected native T1 (ms)			Corrected native T1 (ms)		
	Low	Mean	High	Low	Mean	High
Sydney	965	1014	1062	970	1019	1069
London	951	1012	1072	942	1015	1088
Birmingham	923	960	997	945	975	1005

A low T1 is considered below the values in the *low* column, and high above the values in the *high* column.

**Figure 1:**
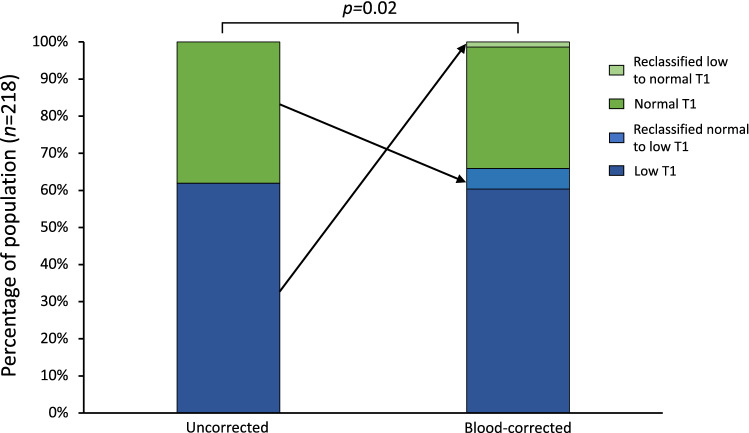
The classification of patients before and following blood-correction. The figure shows the percentage of patients with low T1 (dark blue) and normal T1 (darkt green) before and following blood-correction (135/218, 62% vs. 145/218, 67%). Following blood-correction 3 patients with low T1 was reclassified as normal T1 (light green), whereas 13 patients were reclassified as low T1 (light blue). P-value denotes McNemar’s test.

Without blood-correction, the proportion of FD patients with low myocardial T1 was 135 (62%) out of 218. With blood-correction, 13 previously normal T1 patients (13/83, 16%) were reclassified to low T1, and 3/135 previously low T1 were reclassified to normal T1 resulting in an overall increase in the proportion with low myocardial T1 (135/218, 62% to 145/218, 67%, *p* = 0.02), Fig. 1. None of the healthy volunteers reclassified following blood-correction (3% (3/117) had low T1 before and after).

**Figure 2:**
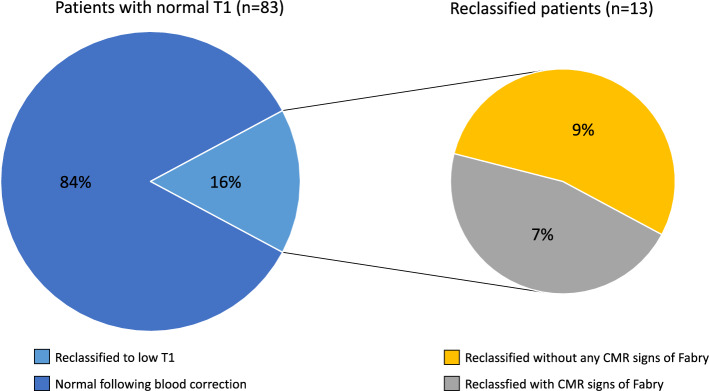
The reclassification of patients with normal myocardial native T1 prior to blood-correction. Following blood-correction, 84% remained normal (blue) whereas 13 (16%) were reclassified as having low myocardial native T1 (light blue). Of these 13, 7 (54%, yellow) had no other CMR signs of Fabry disease (neither LGE or LVH). Displayed in grey are reclassified patients (n = 6, 46%) with other CMR signs of Fabry (either LGE or LVH).

Table 4 summarizes the proportion of FD patients with other CMR signs of cardiac involvement (LVH + /-LGE). Of the patients reclassified as having low T1, 7/13 (54%) did not have any other CMR signs of cardiac involvement of Fabry disease, defined as the presence of LGE or LVH. The remaining 6/13 (46%) of reclassified patients did have LGE and/or LVH, and thus would have been identified as having cardiac involvement regardless of native myocardial T1 value, Fig. 2.

**Table 4:** Number and percentage of LVH/LGE + in uncorrected vs. blood corrected T1.

	Uncorrected	Corrected
Normal T1	14 (14/83, 17% LVH/LGE +)	10 (10/73, 14% LVH/LGE +)
Low T1	97 (97/135, 72% LVH/LGE +)	101 (101/145, 70% LVH/LGE +)

By comparison, 3/135 (2%) of patients with low native T1 prior to blood-correction were reclassified to normal T1 following blood-correction. Of these three patients, one had no LGE or LVH, one had limited basal inferolateral LGE and LVH, and one had LVH but no LGE. In total, 2/3 of patients reclassified to normal, there were other signs of FD cardiac involvement. The two patients reclassified to normal but with other CMR signs of cardiac involvement of FD, had a native T1 marginally inside the normal range (1 and 5 ms from the cut-off limit, respectively).

Native T1 did not correlate with the presence of LGE, *r* = 0.42, p = 0.14, nor did blood-corrected native T1, *r* = 0.07, p = 0.87.

### Characteristics of reclassified patients

The 13 patients reclassified to low T1 were 85% female (*n* = 11) and had higher LV T1 blood (1700 ± 80 vs. 1629 ± 89 ms, *p* < 0.01) and RV T1 blood (1679 ± 80 vs. 1604 ± 90 ms, *p* < 0.01) compared to the non-reclassified FD patients. There was no significant difference in uncorrected native myocardial T1 (962 ± 19 vs. 929 ± 63 ms, *p* = 0.07), LV volumes, mass or hematocrit between these two groups (*p* > 0.05 for all).

### Sex differences following blood-correction

In healthy controls from all sites, as expected, myocardial native T1 was different between sexes: 1026 ± 42 ms in females and 1000 ± 36 ms in males, *p* = 0.001. Following blood-correction, this difference disappeared: 1015 ± 36 ms in females and 1003 ± 34 ms in males, *p* = 0.06. The mean sex difference in healthy controls dropped from 26 to 12 ms. In other words, more than 50% of the difference in myocardial native T1 between females and males disappeared, and the magnitude of the remaining difference was very small.

In FD (x-linked), myocardial native T1 was different between sexes: 953 ± 63 ms in females and 907 ± 51 ms in males, *p* < 0.001. Following blood-correction, myocardial native T1 remained different between sexes: 939 ± 55 ms in females and 901 ± 58 ms in males, *p* < 0.001. However, the mean sex difference in FD patients dropped from 46 to 38 ms (17% reduction in difference).

## Discussion

This image-based multicenter, international study demonstrates that blood-correction of native myocardial T1 increases the number of FD patients classified as having low T1, which is an imaging surrogate for sphingolipid accumulation and hence cardiac involvement. The findings also illustrate the impact of post-processing blood-correction, which is clinically feasible across different sites and scanners, and has the potential to be widely used clinically. The use of average LV and RV blood allows for automatic blood pool detection, which further simplifies post-processing of native T1 maps for increased precision.

### Importance of early identification

Cardiac involvement is complex and the leading cause of death in FD patients^3,4^. Initiating treatment early (before the onset of LVH and scarring) is favorable in terms of better outcomes and to avoid irreversible, progressive disease^5,6^. However, identifying early cardiac involvement is challenging. ECG is cheap and widely accessible technique with parameters of LVH showing alterations in early adulthood^16^. However, low native T1 has been shown to precede the development of LVH^10^, thus CMR plays an important role here. CMR can detect cardiac involvement in FD with the unique ability to accurately quantify LVH, identify the presence of focal fibrosis with LGE, inflammation with T2 mapping, microvascular dysfunction using quantitative perfusion mapping^17^, sphingolipid accumulation using native T1 mapping^18^, and now blood-correction of native T1.

Furthermore, with the availability of expensive Fabry specific therapies, the importance of early and correct FD diagnosis has increased. Long-term outcome studies have shown that FD patients can be event free after 10 years of ERT, and ERT does not impact the progression of cardiac involvement once myocardial fibrosis has developed^19,20^. However, it is currently suggested that sphingolipid accumulation, detected as low myocardial native T1^7^, may trigger LVH, with focal and global inflammation progressing to fibrosis^10^. This highlights the importance of myocardial native T1 as a biomarker in FD. Blood-corrected native T1 maps may lead to earlier detection of sphingolipid accumulation, but this, and the clinical relevance, as well as the potential to be a therapeutic target, needs further investigation with prospective studies. One concern is the potential of misclassifying FD patients as having cardiac involvement, a false positive. In total, three FD patients were reclassified to normal following blood correction. Two of these had other signs of cardiac involvement (one with LVH and one with LVH and LGE) but their blood-corrected native T1s were only marginally inside the normal range (1 and 5 ms from the cut-off limit, respectively).

### Sex differences in native myocardial T1

FD is a X-linked disorder, and men exhibit cardiac involvement earlier than women sometimes due to random X-chromosome inactivation, which possibly may lead to faster myocardial sphingolipid accumulation in men with a more marked T1 reduction as time progresses in pre LVH states^10^. In European recommendations, women should not be treated before onset of organ manifestation^21–23^, which likely contributes to that sex differences in treatment of FD patients still exist, as up 35% of females do not receive treatment despite having major organ involvement^24^. This is further underlined by this study: following blood-correction, T1 remained different between the sexes, suggesting sex differences in cardiac FD beyond that amended by T1 blood-correction. However, it is possible that females may benefit more from blood-correction, given that more females were reclassified in our study. This is important since females develop LVH later compared to males^10^, and therefore potentially could be identified as having cardiac involvement earlier by native T1 blood-correction, and thereby be starting treatment earlier.

It has previously been shown that blood-correction both reduces and eliminates sex differences in native myocardial T1 in non-Fabry patients^11^, which was also shown in the cohort of healthy controls in the current study. Sex differences in FD patients were reduced, however remained significant in FD patients following blood-correction, suggesting that there are sex differences in FD patients that are beyond the sex differences accounted for by T1 blood-correction. Interestingly, the reclassified patients had higher blood T1, highlighting a potential mechanism for blood-correction. Blood, and in particular plasma, have a higher T1 and will affect the measurement of myocardial T1 beyond hematocrit^11^. One might speculate that the higher blood T1, due to a larger proportion of blood plasma, increases the native T1 of the myocardium, which is accounted for by blood-correction, explaining why particularly females with higher blood T1 were reclassified. Hematocrit is an important contributor to blood T1 in combination with iron and HDL cholesterol^25^. However, it is unknown if the blood composition of FD patients differs from other patient groups, but there was no difference in hematocrit between the reclassified and other FD patients.

There are several strengths and limitations to our study. The strengths include a rather large cohort of FD patients across several sites, with different scanners thus increasing the generalizability of the findings. However, this study is a single time-point, observational study without follow-up data. This study lacks myocardial biopsies to validate native T1 blood-correction, because biopsies are not a part of routine clinical care. Another limitation is the absence of correlation with clinical information such as cardiac events and other cardiac investigations like electrocardiograms and echocardiograms, which would further elucidate the clinical impact of native T1 blood-correction. However, this was not the scope of the current study, but rather to show the image-based diagnostic impact of blood-correction for classification of cardiac involvement. Further studies should focus on if blood-correction leads to earlier detection and how that influences the decision to initiate treatment, and the clinical outcome of treatment strategies based on blood-corrected native T1.

## Conclusions

In summary, blood-corrected native T1 increases the number of FD subjects with low T1, and this reclassification tracks other markers of myocardial involvement, without reclassifying any healthy controls. Blood-correction decreases the sex differences in this x-linked disease, potentially offering earlier detection, therapy initiation and improved monitoring of treatment and disease progression, but merits further prospective studies.

## Data Availability

The datasets that supports the findings of the current study are not publicly available due to General Data Protection Regulation but are available from the corresponding author on reasonable request.

## Abbreviations

BSA: Body surface area
CMR: Cardiovascular magnetic resonance
ERT: Enzyme replacement therapy
FA: Flip angle
FD: Fabry disease
FOV: Field of view
GLS: Global longitudinal strain
LV: Left ventricle
LVH: Left ventricular hypertrophy
LGE: Late gadolinium enhancement
MOLLI: Modified Look-Locker inversion recovery
OCT: Oral chaperone therapy
SD: Standard deviation
SSFP: Steady state free precession
TE: Echo time
TR: Repetition time
